# Microfluidics Formulated Liposomes of Hypoxia Activated Prodrug for Treatment of Pancreatic Cancer

**DOI:** 10.3390/pharmaceutics14040713

**Published:** 2022-03-26

**Authors:** Vidhi M. Shah, Craig Dorrell, Adel Al-Fatease, Brittany L. Allen-Petersen, Yeonhee Woo, Yuliya Bortnyak, Rohi Gheewala, Brett C. Sheppard, Rosalie C. Sears, Adam WG. Alani

**Affiliations:** 1 Department of Pharmaceutical Sciences, College of Pharmacy, Oregon State University, 2730 South Moody Avenue, Portland, OR 97201, USA; shahv@ohsu.edu (V.M.S.); afatease@kku.edu.sa (A.A.-F.); rosalynn.woo@gmail.com (Y.W.); bortnyak.yuliya@gmail.com (Y.B.); 2 Brenden-Colson Center for Pancreatic Care, Oregon Health and Science University, 3181 Southwest Sam Jackson Park Road, Portland, OR 97239, USA; dorrellc@ohsu.edu (C.D.); sheppard@ohsu.edu (B.C.S.); searsr@ohsu.edu (R.C.S.); 3 Department of Molecular and Medical Genetics, Oregon Health and Science University, 3181 Southwest Sam Jackson Park Road, Portland, OR 97239, USA; ballenpe@purdue.edu; 4 Department of Pharmaceutics, College of Pharmacy, King Khalid University, Guraiger, Abha 62529, Saudi Arabia; 5 School of Medicine, Oregon Health and Science University, 3181 Southwest Sam Jackson Road, Portland, OR 97239, USA; gheewala@ohsu.edu; 6 Department of Surgery, Oregon Health and Science University, 3181 Southwest Sam Jackson Park Road, Portland, OR 97239, USA; 7 Knight Cancer Institute, Oregon Health and Science University, Portland, OR 97120, USA; 8 Department of Biomedical Engineering, School of Medicine, Oregon Health and Science University, Portland, OR 97239, USA

**Keywords:** hypoxia-activated prodrug, liposome, drug delivery, organoids, pancreatic cancer

## Abstract

Pancreatic ductal adenocarcinoma (PDAC) presents as an unmet clinical challenge for drug delivery due to its unique hypoxic biology. Vinblastine-N-Oxide (CPD100) is a hypoxia-activated prodrug (HAP) that selectively converts to its parent compound, vinblastine, a potent cytotoxic agent, under oxygen gradient. The study evaluates the efficacy of microfluidics formulated liposomal CPD100 (CPD100Li) in PDAC. CPD100Li were formulated with a size of 95 nm and a polydispersity index of 0.2. CPD100Li was stable for a period of 18 months when freeze-dried at a concentration of 3.55 mg/mL. CPD100 and CPD100Li confirmed selective activation at low oxygen levels in pancreatic cancer cell lines. Moreover, in 3D spheroids, CPD100Li displayed higher penetration and disruption compared to CPD100. In patient-derived 3D organoids, CPD100Li exhibited higher cell inhibition in the organoids that displayed higher expression of hypoxia-inducible factor 1 alpha (HIF1A) compared to CPD100. In the orthotopic model, the combination of CPD100Li with gemcitabine (GEM) (standard of care for PDAC) showed higher efficacy than CPD100Li alone for a period of 90 days. In summary, the evaluation of CPD100Li in multiple cellular models provides a strong foundation for its clinical application in PDAC.

## 1. Introduction

Pancreatic ductal adenocarcinoma (PDAC), which accounts for more than 90% of pancreatic tumors [1], remains a lethal disease with an overall five-year survival rate of 11% [2]. By the year 2030, it is expected that PDAC will become the second leading cause of cancer-related deaths [3]. The low survival rates are attributable to the nonspecific clinical presentation of the disease, specifically early metastasis and distinctive PDAC biology, where desmoplastic stroma represents anywhere from 15% to 85% of the tumor [4]. In addition, the abundant extracellular matrix in the desmoplastic stroma results in one of the most hypoxic tumors, with median oxygen of 0.3% compared to the normal pancreas with an oxygen level of 7.5% [5]. Currently, gemcitabine (GEM), combined with nab-paclitaxel and FOLFIRINOX (5-fluorouracil, oxaliplatin, and irinotecan), is the standard of care agents used to treat PDAC patients [6,7]. Despite this, PDAC still presents a substantial clinical challenge with a critical need for a new therapeutic modality [8]. To this end, desmoplastic stroma and hypo-vascularity resulting in a hypoxic tumor make PDAC ideal for hypoxia-activated prodrugs (HAPs) [9].

HAPs are selectively activated drugs under hypoxic conditions, sparing the normal cells. To investigate the utility of HAPs in PDAC, we evaluated the activity of an N-oxide derivative of a potent microtubule inhibitor vinblastine (CPD100), which converts to its parent compound under reduced oxygen or hypoxic conditions. However, the compound developed by Cascade Prodrug Inc. (Eugene, OR, USA) displayed a short plasma circulation time, necessitating a formulation platform to increase its circulation and, ultimately, its efficacy. CPD100, a water-soluble compound, was formulated as a sphingomyelin cholesterol liposome using the traditional extrusion platform. The liposomes (CPD100Li) increased the circulation half-life to 5.5 h compared to CPD100 and displayed stability of only three months [10,11]. The limited shelf life, combined with the scale-up challenges with extrusion-based liposomes, presents a limitation for liposomes in general, and in this case, for CPD100Li. Therefore, to achieve a stable and more scalable CPD100Li formulation, the microfluidics platform was optimized and evaluated as part of previous work, which resulted in liposomes comparable to extrusion-based liposomes [12]. The microfluidics platform uses flow dynamics to mix aqueous and organic liquids to encapsulate hydrophilic and/or hydrophobic drugs, resulting in liposomes with reproducible physicochemical characteristics and ease of scale-up.

Moreover, microtubules are dysregulated in cancer, contributing to tumor aggressiveness and resistance to chemotherapy. Microtubule inhibitors such as vinblastine, a potent chemotherapeutic agent with extensive clinical trial data, have never been studied in PDAC, possibly due to their side effects. Using the HAP approach, which renders vinblastine less toxic to normal cells, this work aimed to repurpose an existing FDA-approved agent (vinblastine) and study its combination with GEM as a new therapy for PDAC.

Different HAP formulations have been developed and examined in multiple disease states [13,14,15]. However, this study is the first to evaluate the efficacy of a microfluidics formulated liposomal formulation of a microtubule HAP for treating pancreatic cancer. The first part of the manuscript presents the formulation and stability of freeze-dried liposomes for over 18 months. In the second part, we evaluated the CPD100Li in two human pancreatic cell lines where we observed lower half-maximal inhibitory concentration (IC_50_) of the drug, confirming the hypoxic activation. To replicate tumor 3D conditions, we tested the penetration and fraction of dead and hypoxic cells after the treatment with both CPD100 and CPD100Li. We also investigated the efficacy of CPD100 and CPD100Li in patient-derived organoids obtained through biopsy and screened based on the expression of hypoxia-inducible factor-1 alpha (HIF1A). It is crucial to investigate the efficacy of CPD100 and CPD100Li in an animal model; therefore, we used an orthotopic pancreatic animal model where we compared the efficacy of both formulated and unformulated CPD100 to and in combination with the standard of care gemcitabine (GEM). Overall, the work highlights the first liposomal formulation of a HAP that can be used as a single agent and in combination with GEM to treat pancreatic cancer.

## 2. Material and Methods

### 2.1. Materials

The prodrug, vinblastine-N-oxide monohydrochloride salt (CPD100), was supplied by Cascade Prodrug Inc. (Eugene, OR, USA). The lipid sphingomyelin (SPM) was purchased from NOF America Corporation (White Plains, NY, USA), and cholesterol (Chol) along with calcium ionophore A23187 was obtained from Alfa Aesar (Haverhill, MA, USA). Sephadex PD 10 columns (GE Healthcare Limited) were purchased from VWR (Radnor, PA, USA). The microfluidic polydimethylsiloxane (PDMS) cartridges for liposome preparation were obtained from Precision NanoSystems (Vancouver, BC, Canada). MiaPaCa-2 and Panc-1 cell lines (human pancreas epithelial cell lines) were obtained from Dr. Michelle Ouellette’s lab at the University of Nebraska Medical Center, Omaha, NE, USA. Cell culture supplies including 3D spheroid low attachment plates (Corning #4520), Dulbecco’s modified Eagle medium (DMEM) (Corning), Gibco™ Trypsin-EDTA (0.25%), phenol red, penicillin/streptomycin (Pen/Strep) (Corning), and Dulbecco’s phosphate-buffered saline (DPBS) (Corning) were acquired from Fischer Scientific (Fairlawn, NJ, USA). Fetal bovine serum (FBS) (Seradigm) was obtained from VWR (Radnor, PA, USA). Reagents and disposables for cell culture assays were purchased from VWR (Radnor, PA, USA) or Thermo Scientific (Fairlawn, NJ, USA). Cell Titer-Blue^®^ Cell Viability Assay Kit and CellTox green were obtained from Promega Inc. (Madison, WI, USA). Image-iT™ Red Hypoxia Reagent was obtained from Thermo Fischer Scientific (Waltham, MA, USA). Regents for organoids such as collagenase IV were obtained from Worthington Biochemical Corp. (Lakewood, NJ, USA), DNase I from Sigma Aldrich (St. Louis, MO, USA), and EBSS, and TrypLE from Thermo-Fisher (Fairlawn, NJ, USA). Nude mice for the orthotropic model were obtained from NCI Charles River (Wilmington, MA, USA), and Matrigel was obtained from (Corning) Fischer Scientific (Fairlawn, NJ, USA). Primary antibodies for alpha-smooth muscle actin (α-SMA), HIF1A, and β-actin were purchased from Thermo Fischer Scientific (Fairlawn, NJ, USA). Anti-rabbit 800 (1:25,000) (LI-COR Odyssey (Lincoln, NE, USA) and collagen 1 (Rockland) were purchased from VWR (Radnor, PA, USA), Masson trichrome stain and hematoxylin stain was purchased from Abcam (Cambridge, MA, USA) and Vector Laboratories (Burlington, CA, USA). All other chemicals were obtained from VWR (Radnor, PA, USA).

### 2.2. Preparation of CPD100 Loaded Liposomes

CPD100 loaded liposomes were prepared using sphingomyelin (SPM) and cholesterol (Chol) lipids using a previously published method [12]. Briefly, empty SPM/Chol liposomes were prepared using a benchtop NanoAssemblr™ instrument (Precision NanoSystems Inc., Vancouver, BC, Canada). The lipids (SPM/Chol) were dissolved in 3 mL of ethanol (organic phase), and the aqueous phase was 300 mM magnesium sulfate buffer at pH 4. The liposome was prepared on a disposable PDMS cartridge ™. Based on our previous study, empty SPM/Chol liposomes were prepared at a mixing ratio (aqueous: organic) of 7:1, a flow rate of 10 mL/min, and a lipid concentration of 10 mM [12]. A heating block at 60 °C was used to maintain the SPM/Chol mixture above its lipid transition temperature. Liposomes were purified by passing through a Sephadex PD 10 column, pre-equilibrated with 300 mM sucrose, 3 mM EDTA, and 20 mM HEPES (SHE) buffer (pH 7.5). CPD100 was loaded into the SPM/Chol liposomes using an active drug loading process using ionophore A23187 [10]. Briefly, CPD100 at 5.5 mg/mL was added to the pre-heated empty liposomes at 60 °C. After 10 min of mixing, 33 mM EDTA and 1 μg of A23187/mg of lipid was added to the drug–liposome mixture and stirred in the water bath for 1 h [10]. These conditions resulted in ~65% encapsulation efficiency for CPD100 in liposomes. The drug-loaded liposomes (CPD100Li) were assessed for size and polydispersity index (PDI) using a Zetasizer Nano ZS (Malvern, UK; at 633 nm at 25 °C), and the concentration of CPD100 in the liposomes was determined by reverse-phase high-performance liquid chromatography at a wavelength of 266 nm using methanol: 0.1% formic acid (70:30 *v*/*v*) mobile phase at a flow rate of 0.5 mL/min.

For lyophilization, CPD100 was loaded into empty liposomes prepared using 5% sucrose as a cryoprotectant in the aqueous phase using a previously optimized method [10]. Briefly, CPD100Li was freeze-dried in Wheaton^®^ Vacule^®^ 100 mL vials using a shelf freeze-dryer, FreeZone^®^ Triad™ Freeze Dry System, Labconco, (Kansas City, MO, USA). The liposomes were subjected to a pre-freeze segment (−80 °C for 6 h), followed by a primary drying segment (4 °C/min ramp, hold at −55 °C for 24 h), a secondary drying segment with three ramps and temperature hold: (1) 5 °C/min ramp, hold at −5 °C for 12 h; (2) 0.01 °C/min ramp, hold at 0 °C for 5 h; and (3) 0.01 °C/min ramp, hold at 4 °C. The vials were filled with argon and stoppered for storage at the cycle end. The freeze-dried liposomes were rehydrated with de-ionized water, filtered through a 0.2 μm filter, and assessed for drug loading, size, zeta potential, and PDI using the methods described above over 18 months. The data are presented as Concentration ± SD, Zave ± SD, and PDI ± SD for three replicates.

### 2.3. Cell Proliferation Assay

The effect of freshly prepared CPD100 and CPD100Li on cell proliferation was determined by the CellTiter Blue^®^ assay. Briefly, MiaPaCa-2 and Panc-1 cells were cultured (3000 cells/well) in a 96-well plate using DMEM medium supplemented with 10% FBS and 1% penicillin/streptomycin at 37 °C. Three hours post-seeding, the cells were exposed to DMSO (control), empty liposomes at 12.5 mM (lipid concentration of empty liposomes) (vehicle control), CPD100 (0.02–100 μM) in DMSO, and CPD100Li (0.02–100 μM) and vinblastine (0.01–10000 nM) in DMSO for a total of 72 h. The concentration of DMSO for CPD100 and the vinblastine treatment groups were under 1%. The cells were cultured for 18 h under normoxia 20% and hypoxic 0.1% (*v*/*v*). Post 18 h of incubation, the plates were incubated under 20% O_2_ for the remaining 54 h. After 72 h, the treated cells were incubated for 2 h at 37 °C with 20 µL of the CellTiter Blue^®^ reagent followed by a measurement of the fluorescence intensity at 560_Ex_/590_Em_. All experiments were performed in quadruplicate, and the data are presented as mean IC_50_ ± SD.

### 2.4. 3D Spheroid Assay

Panc-1 cell spheroids were initiated by seeding 10,000 cells/well in 100 µL medium in a 96-well low attachment spheroid plate and allowed to grow and form spheroids for 48 h. After 48 h, 200 µL of fresh media was added before the drug treatment. The spheroids were then treated with CPD100 (2, 20, 200, 2000, and 20,000 nm) and freshly prepared CPD100Li (2, 20, 200, 2000, and 20,000 nm) for an additional 72 h. At the end of this, spheroids were assayed for apoptosis using the CellTox green at a dilution of (1:2000) and hypoxic cell population using Image-iT™ Red Hypoxia Reagent at a concentration of 5 μM. The spheroids were imaged using an Evos Imaging System. Data are presented as % apoptotic and % hypoxic population at one day and seven days.

### 2.5. Pancreatic Organoid Initiation, Passage, and Treatment

Human PDAC organoid lines were generated at the Brenden-Colson Center for Pancreatic Care (BCCPC) from biopsies collected from consenting patients at the Oregon Health and Science University (OHSU) hospital. This study was approved by the OHSU Institutional Review Board (protocol #3609). Briefly, biopsies were minced in 2.5 mg/mL collagenase IV + 0.1 mg/mL DNase I in EBSS and digested at 37 °C for 30 min. The digested cell suspension was then filtered (40 µm), and the remaining tissue was incubated again at 37 °C for 10 min in 5 mL trypsin (TrypLE). Following filtration, this TrypLE cell suspension was combined with the first fraction of cells. After two washes and centrifugation for 5 min at 300 g, cells were resuspended in Matrigel and cultured as previously described [16]. Specifically, for culture initiation, 0.1 × 10^6^ cells were embedded per 50 µL Matrigel droplet. To pass the PDAC organoid lines, crowded droplets were dispersed by pipetting and incubation for 10 min in TrypLE to generate organoid fragments. After washing as described above, these were re-seeded in new droplets at a 1:4 or 1:6 dilution and allowed to re-grow. For drug treatments, a single crowded 50 µL droplet was used to seed twenty 10 µL droplets (1:4 dilution) each in a separate well of a 96 well plate. Before drug administration, these new cultures were allowed to rest for 48 h to recover and reorganize their 3D structure. The patient-derived tumor organoids were screened based on levels of HIF1A to isolate two organoids expressing high and two expressing low HIF1A levels. All four organoids were treated with either CPD100 or freshly prepared CPD100Li at (0.02 nM–20,000 nM). The data are presented as % cell viability ± SD for *n* = 4 replicates.

### 2.6. In-Vivo Orthotopic Model

Female 6-week-old nu/nu mice were obtained from NCI Charles River. All animal studies were performed under an animal care and use protocol approved by the Oregon Health and Science University institutional animal care and use committee. (Protocol number: TR01_IP00001660 date of approval: December 21, 2021, type of review; Three-Year Renewal.) Briefly, 40 µL of Panc-1 cell solution (1 × 10^6^ cells in 1:1 mixture of serum-free media and Matrigel) was injected into the tail of the mouse pancreas under isoflurane anesthesia. Fourteen days post-inoculation, mice were divided into the treatment groups once the tumors reached 4–5 mm in diameter in the pancreas, confirmed through ultrasound imaging. The treatment groups (four mice per group) consisted of the control (saline), CPD100 (40 mg/kg weekly, i.v; total 8 doses) [10,11], CPD100Li (40 mg/kg weekly, i.v; total 8 doses), gemcitabine (GEM) (100 mg/kg biweekly, i.p; total 6 doses) [17], CPD100Li + GEM (40 mg/kg weekly, i.v; +100 mg/kg biweekly, i.p; 8 doses and 6 doses respectively), and vinblastine (5 mg/kg, weekly, i.v;). The animals were monitored for tumor area using VEVO 2100 ultrasound (FUJIFILM Visual Sonics Inc. Toronto, Canada) until death or morbidity (i.e., marked decrease in body weight and/or tumor diameter greater than 1 cm). At the end of the experiment, mice were euthanized and examined for tumor metastasis to various organs, and the tumor was collected for analysis, including; weight, hematoxylin, and eosin (H&E), trichrome staining, immunohistochemistry, and western blot analysis. Data are presented as % changed in the tumor area, which was calculated by normalizing the tumor area to day 0 tumor measurements and percent survival ± SD as a function of time, tumor weight ± SD, and normalized body weight ± SD as a function of days.

### 2.7. H&E-Trichrome Stains and Immunoblots

Tumor tissues were formalin-fixed (VWR, Radnor, PA, USA) and embedded in paraffin. Six-µm sections were prepared for Masson’s trichrome (Abcam) and hematoxylin and eosin (H&E) (vector laboratories) as per the manufacturer’s protocols. Images for histology and trichrome staining were taken using an EVOS microscope. Masson’s trichrome was quantified using user-defined macro in ImageJ 1.51j8 software, National Institutes of Health, USA.

Post-treatment tumor tissue was collected, and protein lysates were obtained for western blot analysis. Briefly, the protein lysate was processed on SDS-polyacrylamide gel electrophoresis and transferred to the nitrocellulose membrane. Membranes were then incubated overnight at 4 °C with mouse anti-α-SMA antibody (1:1000), mouse anti-HIF1A antibody (1:1000), mouse anti-vimentin antibody, and mouse anti-GAPDH antibody (1:5000) followed by incubation with secondary antibody anti-rabbit 800 (1:25,000) (LI-COR Odyssey, Lincoln, Nebraska USA). A LI-COR Odyssey Infrared Imaging System Model 912 was used to visualize the protein bands, then calculated based on densitometry normalized to the GAPDH band using ImageJ 1.51j8 software, National Institutes of Health, USA. All densitometry calculations represent an average of three biological replicates.

### 2.8. Statistical Analysis

Non-linear regression analysis was used to determine the half-maximal inhibitory concentration (IC_50_) for the cell proliferation assay, and one-way ANOVA with post-hoc Tukey HSD analysis (*p*  <  0.05) was used to determine the statistical significance among the IC_50_ values. Using a Student’s *t*-test, the statistical significance was evaluated between dead and hypoxic cells for 3D spheroid studies, and cell viability for CPD100 and CPD100Li treated pancreatic organoids. Statistical significance for tumor area and densitometric analysis was determined using a one-way ANOVA with post-hoc Tukey HSD analysis, where ns (*p* > 0.05), * (*p* ≤ 0.05), ** (*p* ≤ 0.01), *** (*p* ≤ 0.001), and **** (*p* ≤ 0.0001) represent the statistical significance compared to the control group. For western blot densitometry analysis, values were normalized to each experiment’s loading control (GAPDH) and the no-treatment control. The GraphPad Prism 9 software package (GraphPad Software, Inc., La Jolla, CA, USA) was used to perform all the statistical analyses.

## 3. Results

### 3.1. Preparation of CPD100 Loaded Liposomes

SPM/Chol liposomes were prepared using microfluidics at MR of 7:1, FR of 10 mL/min, and a lipid concentration of 10 mM. The concentration of CPD100 loaded in the liposomes was 3.55 ± 0.28 mg/mL, corresponding to an encapsulation efficiency of 65% (Table 1). The concentration of the freeze-dried formulation was 3.27 ± 0.14 mg/mL, which was within the 10% acceptable shelf-life guidelines. The PDI values for both preparations were less than 0.25 (Table 1), suggesting that the liposomes were monodispersed (Figure S1). The zeta-potential of the freeze-dried CPD100Li was 1.68 ± 0.44 mV, which aligns with the zeta-potential of freshly prepared CPD100Li −1.14 ± 0.06 mV [11].

### 3.2. Cell Proliferation Assay

**Figure 1:**
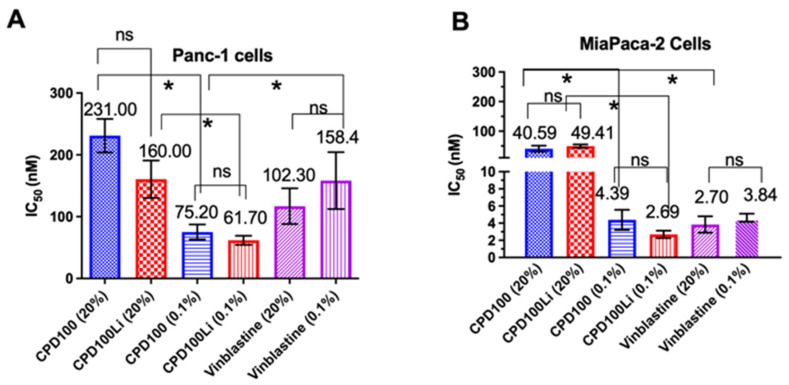
In vitro antiproliferative efficacy of CPD100 and CPD100Li in the PDAC cells lines. In vitro efficacy of CPD100, CPD100Li, and vinblastine (parent compound) under 20% and 0.1% oxygen in the Panc-1 (**A**) and MiaPaCa-2 (**B**) human pancreatic cancer cell lines measured using the CellTiter Blue^®^ assay. Data are represented as the mean ± SD of four replicates. ns (*p* > 0.05), and * (*p* ≤ 0.05), represent the statistical significance compared to the control group by one-way ANOVA.

CPD100 and CPD100Li were tested for their cell proliferative activity under normoxic (20%) and hypoxic (0.1%) conditions in two pancreatic cell lines (Panc-1 and MiaPaCa-2). As anticipated, there was a significant decrease in the IC_50_ values of CPD100 and CPD100Li under hypoxic conditions. The IC_50_ for CPD100 at 20% oxygen was 231 ± 27 nM and 41 ± 10 nM, and at 0.1%, oxygen was 75 ± 12 nM and 4 ± 1 nM in Panc-1 and MiaPaCa-2, respectively. For CPD100Li, it was 160 ± 30 nM and 49 ± 6 nM at 20% oxygen and 62 ± 7 nM and 3 ± 0.4 nM at 0.1% oxygen in Panc-1 and MiaPaCa-2, respectively (Figure 1A,B). Low IC_50_ value at 0.1% oxygen can be explained by the hypoxia activation of CPD100 to the parent molecule vinblastine. There was no statistical difference between the IC_50_ of the prodrug and the lipid encapsulated prodrug as a function of oxygen, confirming that the lipid formulation does not alter the cytotoxic property of CPD100. We also observed no effect of empty liposomes on both cell lines, which confirmed the non-toxic nature of the vehicle. In contrast, vinblastine did not show a difference in IC_50_ as a function of oxygen levels in both cell lines, but MiaPaCa-2 cells were ~19 fold more sensitive to vinblastine than Panc-1 cells (Figure 1A, B). The higher IC_50_ in Panc-1 cells aligns with the anti-proliferation data observed for other chemotherapeutic agents, attributed to the efflux mechanisms and/or chemoresistance in the Panc-1 cell line [18].

### 3.3. 3D Spheroid Assay

**Figure 2:**
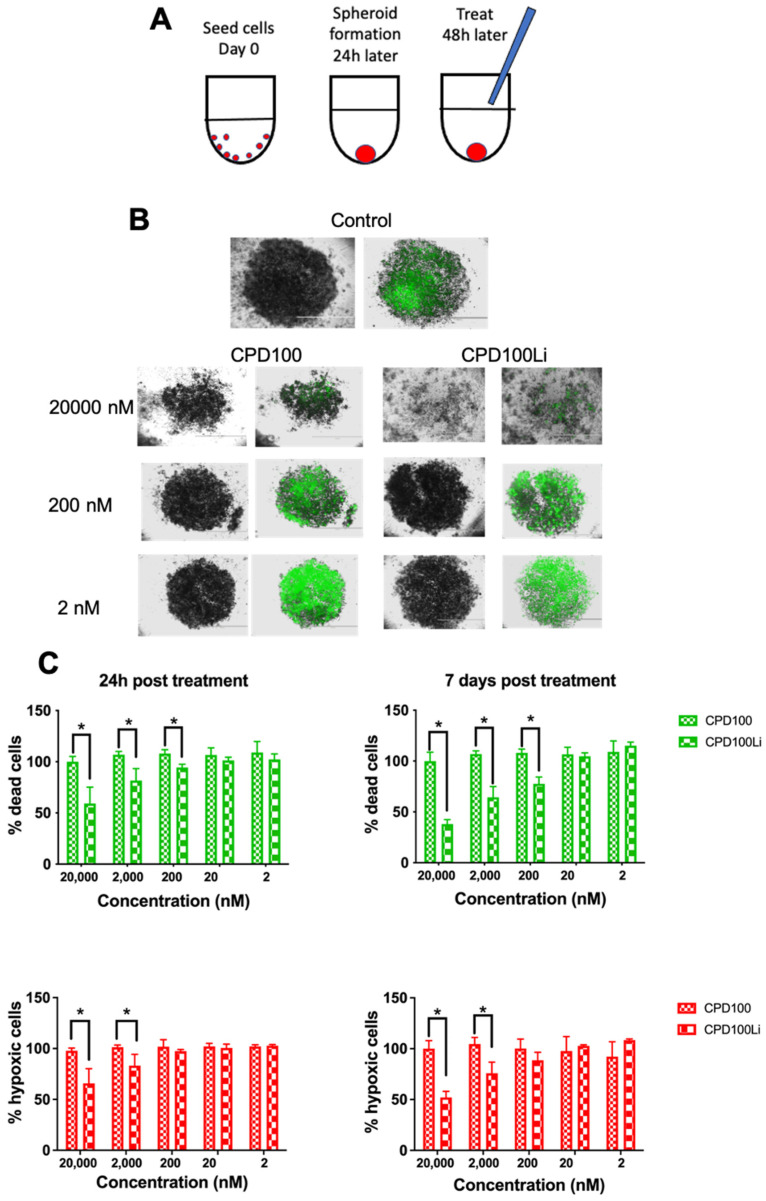
In vitro efficacy of CPD100 and CPD100Li in 3D Panc-1 spheroids. Schematics of spheroid development and treatment (**A**). Representative 4× images of CellTox green staining in response to increasing CPD100 and CPD100Li concentrations for seven days (**B**). Quantification of the percent of hypoxic and apoptotic cells after 24-h and seven days post-treatment with CPD100 and CPD100Li (**C**). Data are represented as mean ± SD of four replicates. * (*p* ≤ 0.05) represents statistical significance compared CPD100Li treated group by the Student-paired *t*-test.

3D spheroids of Panc-1 were developed to investigate the penetration and apoptosis effect of CPD100 and CPD100Li (Figure 2A). Panc-1 cells, when plated at a cell density of 10,000 cells/well, resulted in spheroids with a diameter of ~500 µm at the end of 24 h. Spheroids had lower oxygen levels and thus cell death toward the center compared to the outermost layers, as seen by ImageiT, a hypoxic dye (red), and CellTox green (green), a marker for dead cells (Figure 2B,C). We observed a significant difference in % dead cells at 200, 2000, and 20,000 nM and % hypoxic cells at 2000 and 20,000 nM for CPD100 and CPD100Li treated spheroids at the end of 24 h and 7-day treatment (Figure 2C). A similar trend between hypoxia and dead cells was observed at the end of seven days, suggesting that CPD100Li may be causing cell death in the cells, which are hypoxic (Figure 2B,C). Panc-1 spheroids demonstrated a significantly reduced sensitivity to both CPD100 and CPD100Li compared to cells grown in 2D conditions. Even at the highest drug concentration of 20,000 nM, they did not achieve a 50% reduction in cell viability; thus, IC_50_ could not be estimated for CPD100 and CPD100Li in Panc-1 spheroids. However, we did observe shrinkage and disruption of the spheroids treated with CPD100 and CPD100Li.

### 3.4. Pancreatic Organoid Treatment

**Figure 3:**
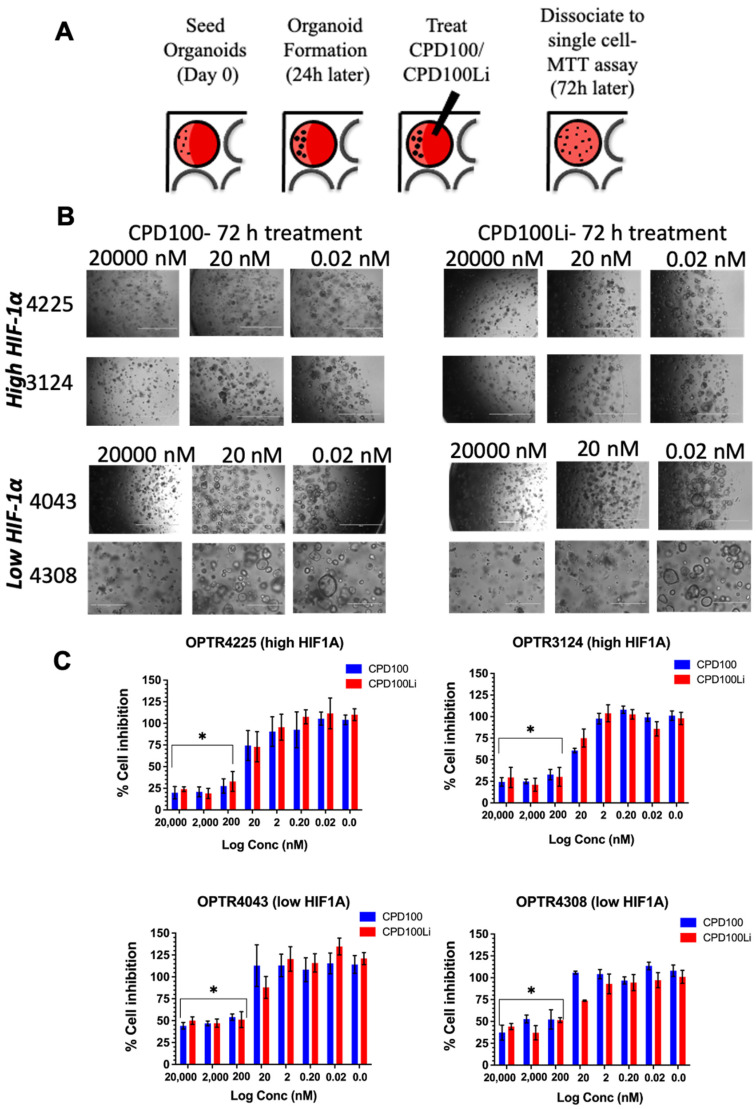
In vitro efficacy of CPD100 and CPD100Li in patient-derived organoids. Schematics of organoid development and treatment (**A**), bright-field images of two high and low HIF1A expressing organoids as a function of three concentrations after 72-h treatment with CPD100 and CPD100Li, scale bar represents 1000 µm (**B**), and % cell inhibition as a function of CPD100 and CPD100Li 72-h post-treatment (**C**). Data represented as mean ± SD of four replicates. * (*p* ≤ 0.05) represents statistical significance compared to the 0.02 nM treated group by the Student-paired *t*-test.

HIF1A levels are known to be regulated by oxygen levels and have also correlated with an unfavorable prognosis in PDAC [19]. Therefore, screening of HAPs in organoids based on *HIF1A* levels could directly correlate between the susceptibility level and patient selection. For example, the OPTR4225 and OPTR3124 organoid lines expressing high levels of *HIF1A* showed about 75% cell inhibition at 200, 2000, and 20,000 nM for both CPD100 and CPD100Li and ~5% cell inhibition at 0.02 nM. In contrast, the OPTR4043 and OPTR4308 organoid cell lines, which expressed low levels of HIF1A, showed around 50% cell inhibition at 200, 2000, and 20,000 nM and about 5% cell inhibition at 0.02 nM (Figure 3B,C). The results suggest that HIF1A levels can correlate with CPD100 and CPD100Li efficacy and that HAP testing in organoid cultures can be further developed to personalize therapy based on tumor oxygenation levels.

### 3.5. In Vivo Orthotopic Model

**Figure 4:**
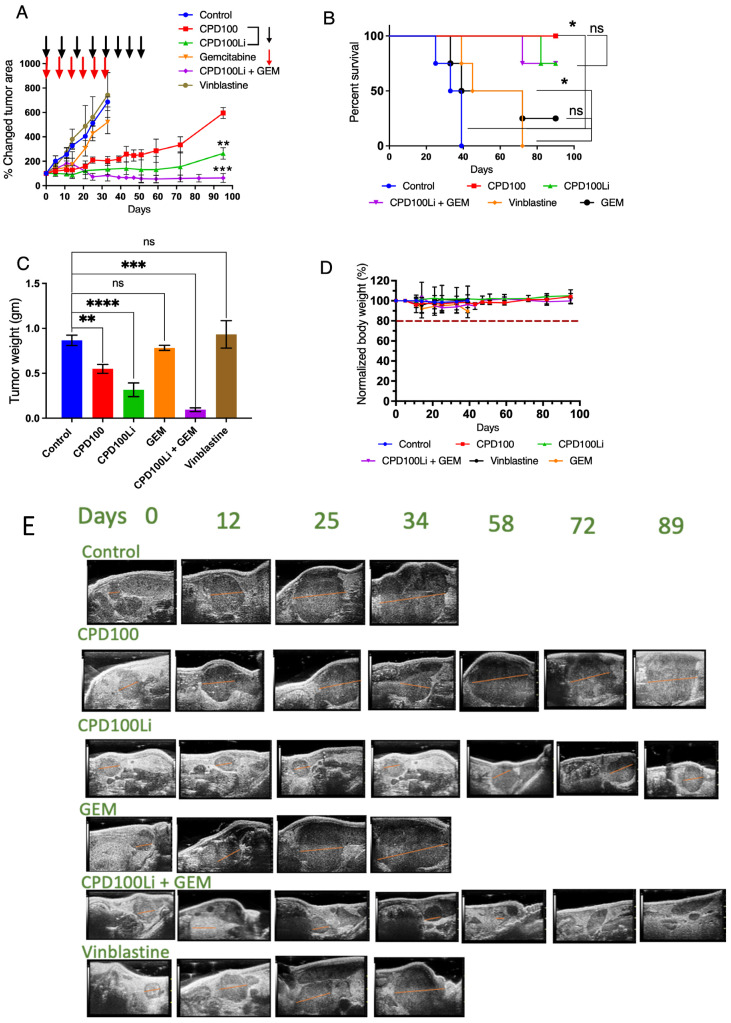
In vivo efficacy of CPD100 and CPD100Li in the Panc-1 tumor model. In vivo efficacy of CPD100, CPD100Li, GEM, CPD100Li + GEM, and vinblastine in PANC-1 orthotopic tumor (**A**), survival curve (**B**), tumor weight post-treatment (**C**), normalized body-weight of mice as a function of days (**D**), representative tumor ultrasound images for the treatment groups; tumor areas were calculated in 3D B-Mode of Vevo 2100 (**E**). ns (*p* > 0.05), * (*p* ≤ 0.05), ** (*p* ≤ 0.01), *** (*p* ≤ 0.001), and **** (*p* ≤ 0.0001) represents statistical significance compared to the control group by one-way ANOVA.

We developed an orthotopic pancreatic cancer model in nude mice and studied the efficacy of CPD100, CPD100Li, GEM, and vinblastine (Figure 4A and Figure S2). Control, GEM, and vinblastine mice were euthanized at a predetermined endpoint as per the animal protocol mentioned in the method section, as shown in the Kaplan–Meier curves and analyzed using the log-rank (Mantel-Cox) method (in-built analysis in GraphPad Prism 9 (GraphPad Software, Inc., La Jolla, CA, USA) software package. The control group had a median survival of 36 days compared to 55 days for vinblastine-treated animals and 58 days for gemcitabine-treated animals (Figure 4B). For the animals treated with CPD100, no deaths were seen until 95 days, indicating that the tumor burden was well tolerated. We did see one death each for CPD100Li treated animals at day 82, and for CPD100Li + GEM treated animals at day 72 (Figure 4B), but the cause of death is unknown and was not the tumor burden as the tumors constantly measured as small for both groups. The final tumor weights of mice treated with CPD100Li and CPD100Li + GEM showed a statistically significant decrease at *p* < 0.05 in (Figure 4C) compared to the control mice, GEM, and vinblastine (Figure 4A,B,E), respectively, consistent with the previous findings in different animal models [10,11]. Moreover, when administered to pancreatic tumor mice, vinblastine, the parent compound of CPD100, showed no significant difference in tumor volume and survival compared to the control animals. There was no evidence of drug-induced toxicity or metastasis as all mice gained weight equally in all groups (Figure 4D).

### 3.6. H&E-Trichrome Stains and Immunoblots

**Figure 5:**
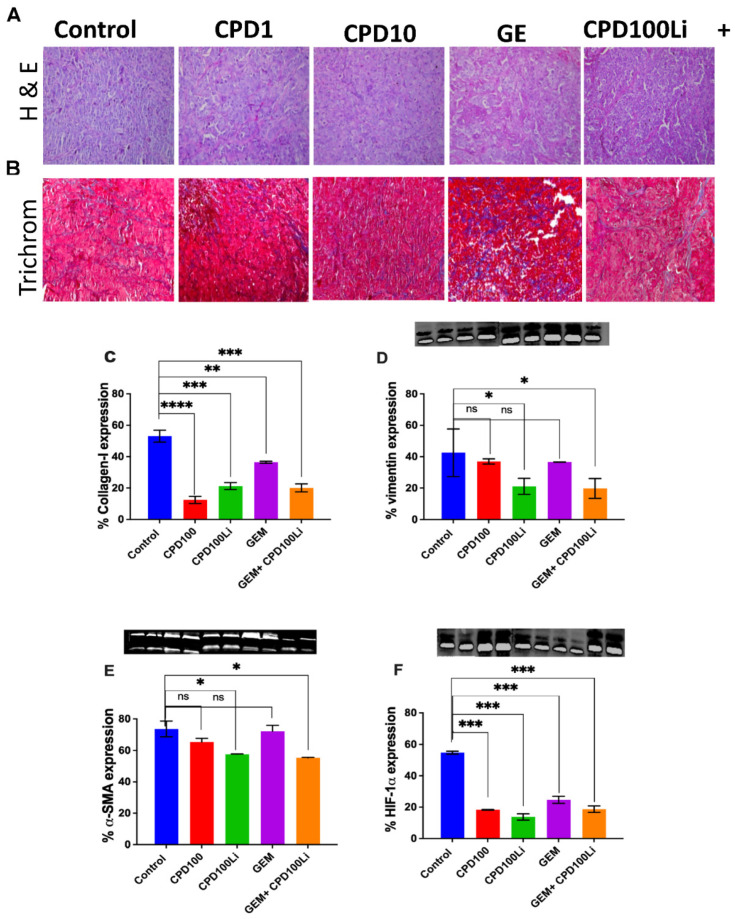
H&E-trichrome stains and immunoblots. Representative (**A**), H&E and (**B**), Masson’s trichrome stain of tumors from the treatment groups: control, CPD100, CPD100Li, GEM, CPD100Li + GEM, scale bar represents 200 µm (**C**), % collagen quantified in trichrome stain using ImageJ and immunoblots of (**D**), % vimentin expression (**E**), % α-SMA expression and (**F**), % HIF1A expression. Error bars are standard deviations. ns (*p* > 0.05), * (*p* ≤ 0.05), ** (*p* ≤ 0.01), *** (*p* ≤ 0.001), and **** (*p* ≤ 0.0001) represent the statistical significance compared to control group by one-way ANOVA.

H&E and Masson’s trichrome staining revealed collagen deposition within the tumor tissue of all the treatment groups (Figure 5A). ImageJ analysis revealed a significant increase in collagen deposition (blue staining) in response to GEM compared to the vehicle and control groups. Additionally, western blot analysis showed that αSMA and vimentin expression were significantly lower in the CPD100Li and CPD100Li + GEM group than in control, CPD100, and GEM (Figure 5C,D), confirming decreased activation of fibroblasts in the liposomal and combination group.

## 4. Discussion

Pancreatic cancer is a deadly disease due to its distinct pathological barriers such as desmoplastic stroma and hypoxic microenvironment, increasing aggressiveness and hindering drug delivery to pancreatic tumor cells [20,21]. The barriers suggested depletion of the stroma as a breakthrough approach to ameliorate this deadly disease only to observe failed preclinical and clinical trials, which had to be terminated due to increased aggressiveness of the disease [21,22]. In the current scenario, where different treatment approaches have not been able to increase the survival rates of pancreatic patients, a liposomal formulation of HAP is a novel approach to treat highly hypoxic pancreatic cancer. Furthermore, numerous studies in multiple model systems have confirmed the activity of CPD100 as a function of oxygen [11], making it an attractive therapeutic for PDAC.

Here, we have described for the first time a liposomal formulation of hypoxia-activated prodrug CPD100Li prepared using the microfluidic platform to treat solid pancreatic tumors [10,11]. CPD100Li was freeze-dried due to the instability of liposomes in solution form. The low stability of liposomes in the solution form can be attributed to changes in size distribution due to the degradation of lipids and leakage of encapsulated material due to a decrease in permeability of the lipid membrane. Therefore, the liposome size and PDI, and encapsulation efficiency were assessed for CPD100Li over 24 h and 18 months (Table 1 and Figure S1), with a kinetic drug release primarily in the first 6 h, as shown in our previous publication [12]. The formulation is easily scalable, reproducible, and has a shelf life of over 18 months when freeze-dried (Table 1). The hypoxia-mediated activation of CPD100 and CPD100Li has been studied in Panc-1 and MiaPaCa-2 cell lines (Figure 1), which express both epithelial and mesenchymal markers, allowing them to be characterized as epithelial-mesenchymal cells [23]. Epithelial-mesenchymal transition (EMT) has been shown to contribute to drug resistance in pancreatic cancer and is characterized as an important mechanism by which the epithelial cancer cells acquire the malignant phenotype. Panc-1 is highly prone to epithelial-mesenchymal transition and has been shown to have no E-cadherin expression, and the lack of E-cadherin expression is linked to a lower survival rate in several tumor types. Both cell lines showed higher susceptibility to CPD100 and CPD100Li at lower oxygen levels, consistent with the activity of other HAPs (Figure 1). Moreover, the efficacy of the drug molecule in both hypoxic and normoxia conditions remained unaffected by the liposomal formulation (Figure 1).

Despite the significant achievements to cancer drug development due to in vitro drug testing in the monolayer culture, it lacks specific intrinsic characteristics of in vivo environments such as cell packing and arrangement, growth kinetics, and a concentration gradient of the therapeutic agent [24,25,26]. These features play a crucial role in drug testing for pancreatic cancer and can be achieved with 3D spheroids [24]. Moreover, due to its packing characteristics and lack of oxygen diffusion, 3D spheroids create a hypoxic gradient through their layers, which mimics the tumor characteristics, ideal for HAP testing [27]. Delivery of the chemotherapeutic is a significant challenge in pancreatic cancer, so we created spheroids of Panc-1 cells to evaluate the penetration and determine our cytotoxic selectivity toward hypoxic cells. CPD100Li at the highest concentration was able to disintegrate the 3D spheroid compared to CPD100 (Figure 2), which may be attributed to better uptake of the lipid vesicle by the Panc-1 cells; this aligns with data for other nanosystems where a size of around 100 nM particles had better spheroid penetration compared to unencapsulated molecules [28,29]. The cell viability of Panc-1 spheroids treated with CPD100 and CPD100Li did not show a decline in the cell viability >50% even at the highest concentration compared to the untreated control (Figure 2). The metabolic activity assay can explain this as we used it to quantify the cell proliferation at the end of treatment, which measured the relative change but not the adjustment in metabolic activity of the spheroid cells on treatment. CPD100Li showed an increased ability to penetrate both the spheroids’ hypoxic and apoptotic cell fraction, which could explain its higher efficacy than CPD100 (Figure 2B). This might be attributed to the lipid–cell interactions, resulting in the disruption of spheroids at higher concentrations, as seen in Figure 2B [30]. The ImageiT, a hypoxic dye (red), and CellTox green (green), a marker for dead cells, added to the cells during spheroid generation, show the fraction of cells that are hypoxic and apoptotic at the initiation and termination of the treatment (Figure 2B, C). In contrast to other published works [31,32] that subjected the spheroids to anoxic conditions, we initiated a spheroid at high cell density to create a pathophysiological hypoxic condition [33]. Generally, the efficacy of drugs tested in 3D spheroids is lower due to increased drug resistance compared to monolayer cultures, but they have been utilized as a model to study the penetration and shrinking ability of drug-loaded nanocarriers [24].

Pancreatic organoids are three-dimensional epithelial cultures derived from primary tissues obtained from patients during surgery or biopsy [34]. The organoids are a novel platform for drug screens as they retain properties of self-renewal, self-organization, genotype, and much of the phenotype of the parent tumor compared to cellular and xenograft models [34,35,36]. Screening of organoids thus reduces the translational gap in the disease by tailoring therapeutics based on gene expressions of the patient tumors [34,35,37,38]. Our screening of patient-derived organoids with CPD100 and CPD100Li showed higher cell inhibition in organoids that expressed higher HIF levels (about 75%) compared to organoids that expressed lower levels (about 50%) (Figure 3C). Even though this observation requires further validation, it is exciting and can provide a patient screening platform for treatment with HAPs depending on the levels of HIF1A.

In addition, we developed an orthotopic animal model consisting of human pancreatic cancer cells implanted directly into the pancreas of immunodeficient mice. The model better represents the human tumor and imitates natural tumor progression with high reproducibility. In our animal models, we compared CPD100 to CPD100Li as we have previous studies showing that the liposomal encapsulation increases the circulation time of CPD100 from a half-life of 26 min to 5.5 h and higher cell penetration along with uptake [11]. We also compared both the treatments to a control (saline-treated), GEM (standard of care chemotherapy), and vinblastine (parent compound). CPD100 is a microtubule inhibitor; we predicted that it might show a similar effect on the stroma as nab-paclitaxel, the nanoformulation that increases the survival of patients with pancreatic cancer by increased GEM efficacy [39]. The importance of additional treatment arms was to show that the liposomal encapsulation of CPD100 markedly improved tumor burden, as seen by the reduction in tumor area and survival (Figure 4A–E and Figure S2). In addition, the CPD100 treated arm showed better tolerance to the tumor burden than the control, vinblastine, and gemcitabine treated animals, as confirmed by the tumor area measurements and survival data. Lower efficacy of CPD100 was anticipated and observed in the tumor model due to short circulation time, and thus the CPD100 + GEM arm was not studied (Figure 4A–C). The CPD100, CPD100Li, and CPD100Li + GEM treated animals survived 95 days compared to the control animals with a median survival of 36 days (Figure 4B). Moreover, none of the animals showed a decrease in weight, suggesting that the doses were not toxic and well-tolerated (Figure 4D). CPD100Li + GEM treated animals also showed significantly decreased α-SMA, collagen, vimentin, and HIF1A expression (Figure 5), suggesting a stromal thinning effect of CPD100Li.

Pancreatic stellate cells (PSCs) are quiescent star-shaped stromal cells with specific characteristics such as vitamin A lipid droplets [40]. During PDAC, PSCs are activated and transformed into a myofibroblast-like phenotype by some risk factors such as hypoxia [40]. Once activated, they lose the lipid droplet and express fibroblast activation proteins: αSMA and vimentin [40]. Activated PSCs display increased proliferative capacity and synthesize abundant extracellular matrix, resulting in a desmoplastic reaction, which results in a barrier to drug delivery [40,41]. Research in the field suggests that the prognosis and progression of the PDAC are dependent on the stroma–collagen architecture (fibrosis) and the resulting tissue tension [42]. However, numerous approaches ranging from targeting the genetic driver of ECM such as transforming growth factor-β (TGFβ) or STAT3 [43] to therapeutic intervention such as matrix metalloproteinase 2 (MMP2) liposome loaded pirfenidone [44] or an angiotensin receptor II inhibitor, losartan [45], which can function as anti-fibrotic agents, have resulted in limited clinical success. We observed that CPD100Li combined with GEM resulted in decreased expression of the α-SMA, collagen, and vimentin levels, suggesting that hypoxia-activated prodrug is worth pursuing as a therapeutic modality for PDAC.

Moreover, HIF1A is one of the primary genes to be activated in response to lower oxygen levels [46], and studies have shown that orthotopic pancreatic tumors exhibit pockets of hypoxia restricted not only to the center of the tumor but are also spread randomly throughout the tumor and its periphery, thus mimicking the human tumor [47]. HIF1A expression in orthotopic tumors was significantly higher in the control group than the rest of the treatment groups (Figure 5F); this may be attributed to smaller tumors in the rest of the groups compared to the control. Overall, we have shown that CPD100Li affects re-modulating stroma, which facilitates drug delivery and penetration of gemcitabine in pancreatic tumors, thus increasing the chemotherapeutic efficacy of this agent in pancreatic cancer. This experiment further strengthens the therapeutic effectiveness of CPD100Li to inhibit the growth of one of the most hypoxic and therapeutically challenged tumors.

## 5. Conclusions

We formulated a liposomal formulation of a HAP using the microfluidics platform. The lipid formulated HAP has a shelf life of over 18 months and efficiently delivers chemotherapeutics to pancreatic tumors. Using 3D spheroids and patient-derived organoid culture systems, we have shown a unique way of predicting the sensitivity of the novel delivery systems in pancreatic patients. In addition, remodeling stroma by combining agents can effectively suppress tumor growth in GEM-resistant pancreatic tumor models. Overall, our liposomal HAP strategy shows potential for increasing the clinical outcome of patients with pancreatic cancer and should be investigated further to develop a potent combination therapy.

## Figures and Tables

**Table 1:** The long-term stability of CPD100 liposomes prepared using the microfluidics method.

Formulation	Duration	Concentration (mg/mL)	Encapsulation Efficiency (%)	Zave ± SD (nm)	PDI ± SD
CPD100Li-suspension	1 day	3.55 ± 0.28	65	105 ± 1.2	0.212 ± 0.016
CPD100Li Freeze-dried	18 months @ 4 °C	3.27 ± 0.14	60	95 ± 3.7	0.240 ± 0.019

## Data Availability

All data available are reported in the article.

## Supplementary Materials

The following are available online at https://www.mdpi.com/article/10.3390/pharmaceutics14040713/s1, Figure S1. DLS size measurement of (A) CPD100Li-freeze dried liposomes and (B) CPD100Li-suspension. Figure S2. Individual tumor measurements of animal groups treated with (A) Control; (B) CPD100; (C) CPD100Li; (D) GEM; (E) CPD100Li + GEM; (F) Vinblastine.
