# Supplementary material for: Microfluidics Formulated Liposomes of Hypoxia Activated Prodrug for Treatment of Pancreatic Cancer
Source: Pharmaceutics. 2022 Mar 26;14(4):713. doi: 10.3390/pharmaceutics14040713 (PMC9031349; doi:10.3390/pharmaceutics14040713)
Supplement: Supplementary file 1 [file pharmaceutics-14-00713-s001.zip › pharmaceutics-1603561-supplementary.pdf]

pharmaceutics

MDPI

Supplementary Materials: Microfluidics Formulated Liposomes of Hypoxia Activated Prodrug for Treatment of Pancreatic Cancer

Vidhi M. Shah, Craig Dorrell, Adel Al-Fatease, Brittany L. Allen-Petersen, Yeonhee Woo, Yuliya Bortnyak, Rohi Gheewala, Brett C. Sheppard, Rosalie C. Sears and Adam WG. Alani

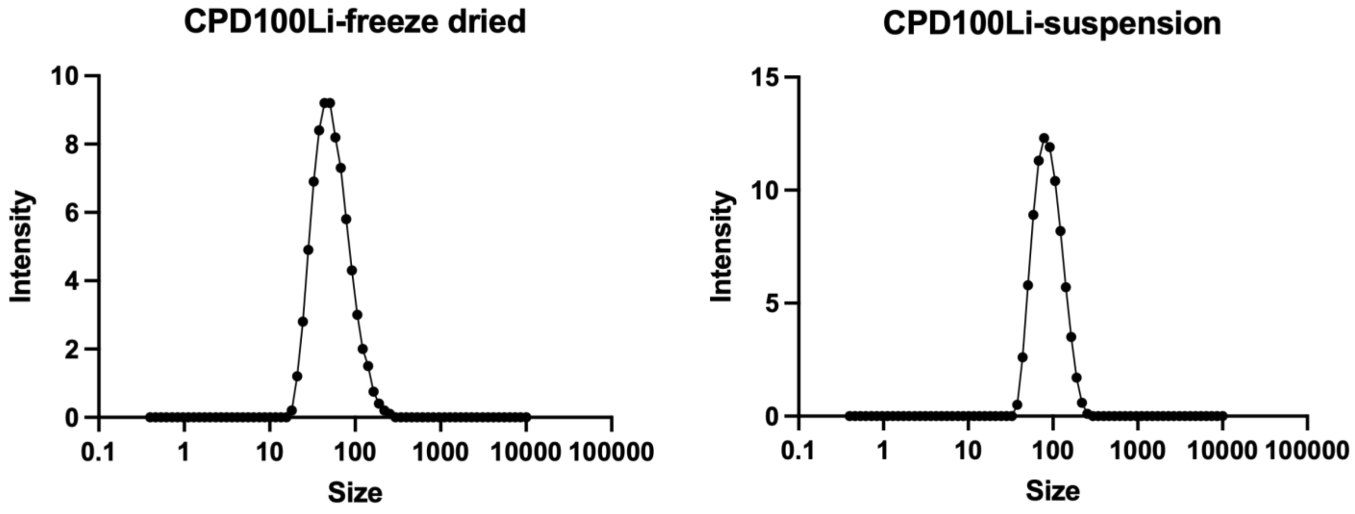

(**A**) (**B**)

Figure S1. DLS size measurement of (**A**) CPD100Li-freeze dried liposomes and (**B**) CPD100Li-suspension.

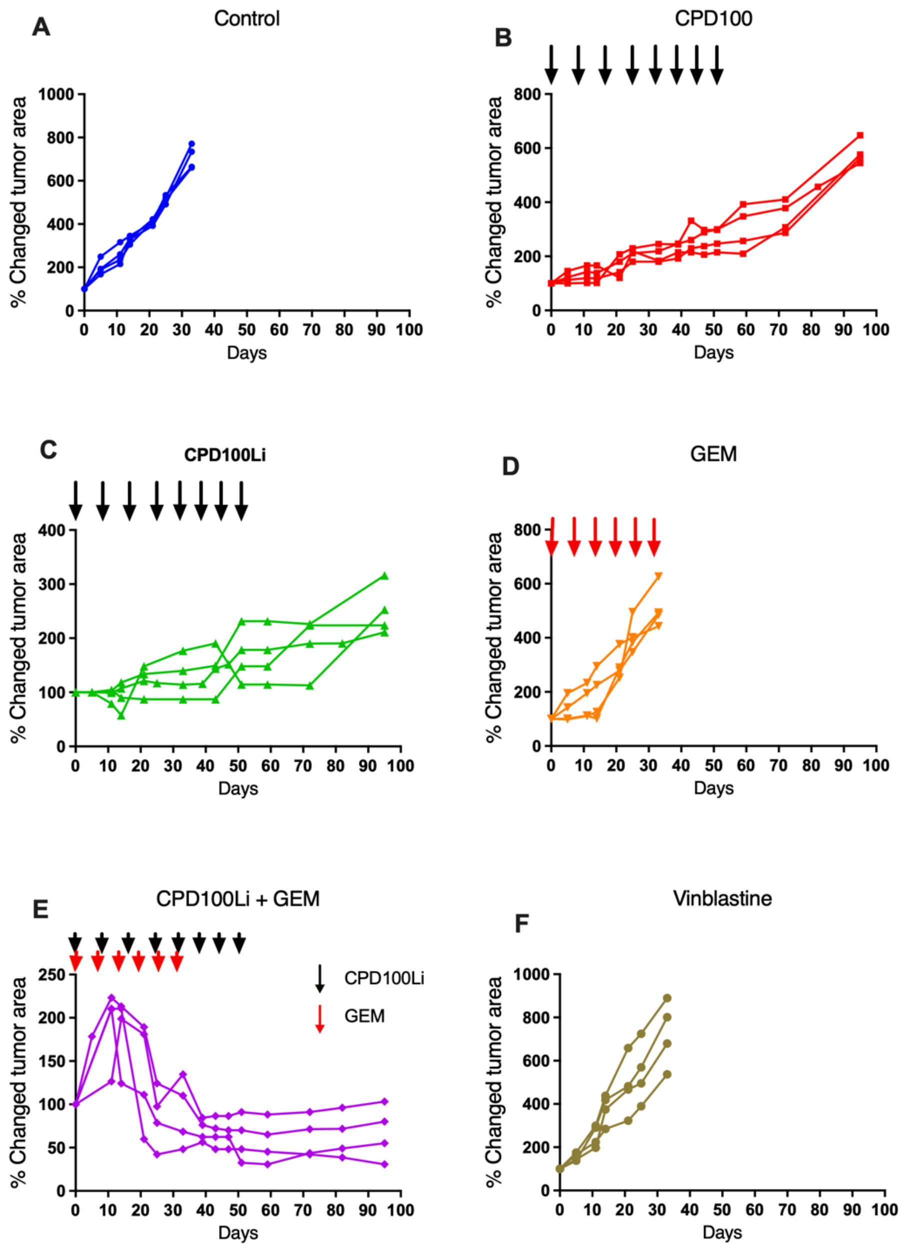

Figure S2. Individual tumor measurements of animal groups treated with (**A**) Control; (**B**) CPD100; (**C**) CPD100Li; (**D**) GEM; (**E**) CPD100Li + GEM; (**F**) Vinblastine.
